# Predictors of depression among patients receiving treatment for snakebite in General Hospital, Kaltungo, Gombe State, Nigeria: August 2015

**DOI:** 10.1186/s13033-017-0132-8

**Published:** 2017-04-13

**Authors:** Abdulaziz Muhammed, Mahmood M. Dalhat, Babalola O. Joseph, Abubakar Ahmed, Patrick Nguku, Gabriele Poggensee, Mukthar Adeiza, Garba I. Yahya, Muhammad Hamza, Zaiyad G. Habib, Abisola M. Oladimeji, Abdulsalam Nasidi, Abubakar Balla, Ibrahim Nashabaru, Nasir Sani-Gwarzo, Ahmad M. Yakasai, Joshua A. Difa, Taiwo Lateef Sheikh, Abdulrazaq G. Habib

**Affiliations:** 1 Nigeria Field Epidemiology and Laboratory Training Program, Abuja, Nigeria; 2 Department of Medicine, Ahmadu Bello University, Zaria, Nigeria; 3 Department of Medicine, Bayero University, Kano, Nigeria; 4 Department of Medicine, University of Abuja Teaching Hospital, Abuja, Nigeria; 5 Nigerian Centre for Disease Control, Abuja, Nigeria; 6 Kaltungo General Hospital, Kaltungo, Gombe State Nigeria; 7 Port Health Services, Department of Public Health, Federal Ministry of Health, Abuja, Nigeria; 8 Public Health and Diagnostic Institute, College of Medical Sciences, Northwest University, Kano, Nigeria; 9 Ministry of Health, Gombe, Gombe State Nigeria; 10 Department of Clinical Services, Federal Neuropsychiatric Hospital, Kaduna, Nigeria

**Keywords:** Depression, Snakebites, Envenomation, General hospital

## Abstract

**Background:**

Snakebite though neglected, affects 5 million people yearly. More neglected is the psychological effect of envenomation. We determined prevalence and pattern of depression among patients admitted into snakebite wards of Kaltungo General Hospital Nigeria, and percentage recognized by clinicians. We also assessed for factors associated with depression.

**Methods:**

In a descriptive hospital based study, we used Patient Health questionnaire (PHQ-9) to make diagnosis of depression among the patients. We reviewed patients’ clinical records to determine clinicians’ recognition of depression.

**Results:**

Of 187 interviews analyzed, 47 (25%) had depression with none recognized by attending clinicians. Patients with snakebite complications (odd ratio [OR] 3.1, 95% CI 1.1–8.5), and previous history of snakebites (OR 2.7, 95% CI 1.1–6.1) were associated with mild depression. Worrying about family welfare (OR 31.5, 95% CI 6.5–152.9), financial loss (OR 14.6, 95% CI 1.8–121.5) and time loss (OR 14.6, 95% CI 1.8–121.5), past history of snakebites (OR 8.3, 95% CI 1.9–36.5) and lower income (Mean difference −25,069 [84 USD], 95% CI 35,509 [118 USD]–14,630 [49 USD]) were associated with severe depression.

**Conclusion:**

A quarter of in-patients of snakebite wards of the general hospital had comorbid depression that went unrecognized. Independent predictors of depression such as past history of snakebite, worrying about relations and having snakebite complications could help clinicians anticipate depression among patients. We recommend training of clinicians in the hospital on recognition of common psychological disorders like depression.

## Background

Snakebite though neglected, is an important cause of both mortality and morbidity. About 5 million people are estimated to be bitten by snakes every year, out of which 125,000 die and 400,000 permanently disabled or disfigured [1]. The burden is said to be highest in South Asia, Southeast Asia, and sub-Saharan Africa. Recently it has been estimated that between 10,000 and 100,000 snakebite envenoming occur in the West African region, with an incidence of 9–90/100,000 persons per year and an estimated 1000–10,000 deaths with a mortality rate of 0.5–6.0/100,000 persons per year [2].

More neglected than snakebite itself, is the psychological consequences resulting from experiencing an envenomation [3]. Snakebites are usually sudden and perceived as life threatening or at least a very stressful event causing severe disruption to the activities of daily living of the victim. Considering the fact that most victims of snakebite in West Africa are rural farmers who survive on subsistence farming, all of the above could serve as risk factors for the development of psychological disorders. Very few studies have been done on the development of psychiatric disorders among snakebite victims in general, and Nigeria in particular. In a study of adult patients who were victims of snakebite, the mean score on Beck’s depression inventory was significantly higher among victims of snakebites than among controls. The authors concluded that snakebite causes significant ongoing psychological morbidity, a complication not previously documented [4].

We set out to determine the prevalence and pattern of depression among snakebites victims admitted into the snakebite wards of the General Hospital Kaltungo. We identified the proportion recognized by clinicians and assessed for factors associated with depression among the patients. Finally, we determined predictors of depression among the victims in other to improve the mental health system at the general hospital Kaltungo.

We hypothesized that there are no predictors of depression among patients admitted into General hospital Kaltungo.

## Method

### Study setting

The study was conducted in General Hospital Kaltungo, which host the regional snakebite reference center in Kaltungo Local Government Area of Gombe State, North-Eastern Nigeria. Kaltungo is located within the Sudan savannah vegetation of the Benue river valley known for carpet vipers’ envenomation. The reference center has a 22 and 16 bed capacity male and female wards respectively.

### Study design

We conducted a hospital based cross sectional study, with exploratory component.

### Study population

The study population consisted of patients admitted into the snakebite ward of General Hospital Kaltungo, Gombe State during a 1-month period from the 4th of August to 4th of September 2015.

### Inclusion criteria

1. Persons admitted into the male and female snakebite wards of General Hospital Kaltungo between 4th of August and 4th of September 2015.
2. Patients aged 18 years and above.

### Exclusion criteria

1. Past history of depression prior to presentation in the snakebite ward for envenomation.
2. Patients too ill to answer research questionnaire.

### Sample size determination

We calculated the minimum sample size required for the study using the Leslie and Kish formula for estimating sample size for cross-sectional study [5].

$$ {\text{n}} = \, \left( {{\text{Z}}\alpha^{ 2} {\text{pq}}} \right)/{\text{d2}} $$

where n = Minimum sample size, Zα set at 5% significant level = 1.96, p = estimates of proportion of study population with depression. We used a prevalence of 19% (0.19) symptoms of depression among persons with snake bite envenomation in a study on delayed psychological morbidity associated with snakebite envenoming [4], d = level of precision set at 5%

$$ {\text{q }} = { 1} - {\text{p }} = \, 0. 8 1 $$

The calculated sample size was 236.

We adjusted the calculated sample size for small population size. The General Hospital Kaltungo medical records indicated an average of 300 admissions per month at that time of the year. Therefore, using the formula for finite population correction,

$$ nf = \frac{n}{1 + (n)/(N)} $$

where: nf = the desired sample size when population is less than 10,000; n = the desired sample size when the population is more than 10,000; N = the average number of patients admitted per month in the male and female snakebite wards of the general hospital Katungo (N = 482) as at the time of study.

Thus

$$ nf = \frac{236}{1 + (236)/(300)} $$

Nf = 132

For this study we recruited 190 respondents.

### Sampling technique

From 4th of August 2015 we consecutively recruited all snake bite patients admitted into the male and female snakebite wards of General Hospital Kaltungo who met our recruitment criteria till the achievement of our required sample size.

### Study instruments

We designed a structured interviewer administered questionnaire to obtain the socio-demographic characteristics and past history of depression among the respondents. The ninth revision of the Patient Health Questionnaire (PHQ 9) was used in assessing depression. The PHQ-9, in its original form, is a self-administered nine-item version of Primary Care Evaluation of Mental Disorders (PRIME-MD) specific to depression [6, 7]. For this study we used trained interviewers for the administration of the PHQ-9 to respondents due to low literacy rate in the area. PHQ-9 has a good test–retest reliability, criterion validity [8], and construct validity [7]. Its capacity to detect meaningful change and shorter time of administration promote its choice for our survey. The questionnaire consists of nine questions scored on a likert scale of 0–3. The aggregate score per respondent could ranges from 0 to 27 based on the answer to each of the nine questions. A cut-off score for minimal (0–4), mild [5–9], moderate [10–14], moderately severe [15–19], and severe (≥20) depression has been suggested [8]. A score of ≥10 has been shown to have an 88% sensitivity and 88% specificity for major depression in a general medical population [8]. For this study a score of 0–4 was defined as no depression, 5–9, as mild depression, 10–14 as moderate depression and >14 as severe depression. The PHQ-9 has been validated for use among Nigerian subjects [9] and has been used to diagnose mental disorders among patients at primary health care in Nigeria [10].

### Data collection and procedure

We recruited as data collectors, five residents of the Nigerian Field Epidemiology and Laboratory Training Program [11] with extensive experience with data collection from prior activities. They were trained for a period of 5 days, prior to the onset of the study, on the use of the study questionnaires and interview techniques. Data collection took place between 4th of August and 4th of September 2015. The average duration of interview was 20 min.

### Data management and analysis

Data were entered into Epi info 3.3.2, cleaned and edited for inconsistencies before analysis. We summarized our findings using frequencies, means (with standard deviation) and proportions. We used Odds Ratio (OR) with 95% Confidence Interval (95% CI) to check for statistically significant associations and unconditional logistic regression to check for independent predictors of psychological distress.

### Ethical consideration

We obtained ethical approval and permission to conduct this study from the Ethical Committee of the Aminu Kano Teaching hospital, Kano, Nigeria and Ministry of Health, Gombe State. Written informed consent was obtained from study participants. All collected data were archived and protected within the data base of the Nigeria Field Epidemiology and Laboratory Training Program (NFELTP). All snakebite patients involved in the study were treated with anti-venoms after clinical and laboratory evaluation suggested envenomation. A mental health specialist attended to patients with clinically significant psychological disorders. Participation of patients in the study was based on their willingness to be involved and were free to exit the study at any stage without compromise to their standard of care.

## Results

We analyzed 187 of the 190 patients interviewed. Three were disqualified because the patients left before concluding the interview. The mean age and standard deviation of the patients was 33.8 ± 14.1 years and age ranged from 18 to 80 years.

Of the 187 patients, 136 (73%) were between the age group 20–49 years, 154 (82%) were males and 109 (58%) had no formal education. Most of the patients 134 (71%) travelled more than a 100 km before getting to the hospital with 89 (48%) spending over 8 h before reaching the hospital (Table 1).

**Table 1:** Socio-demographic characteristics of patients admitted into snakebite wards, General Hospital Kaltungo, 2016 (n = 187)

Socio-demographic variables	Frequency	Proportion (%)
Age (years)		
<20	17	9.1
20–49	136	72.7
>49	34	18.2
Gender		
Male	154	82.4
Female	33	17.6
Education		
Formal	78	41.7
No formal education	109	58.3
Occupation		
Farming	101	54.0
Cattle rearing	49	26.2
Student	12	6.4
Others	25	13.4
Average monthly income (dollars)		
Rich (>150)	48	25.7
Average (70–150)	45	24.1
Poor (40–69)	49	26.2
Very poor (<40)	45	24.1
Distance to health facility (Km)		
<50	21	11.2
50–99	32	17.1
100–149	38	20.3
150–199	60	32.1
>200	36	19.3
Time taken to reach health facility (h)		
<4	54	28.9
4–8	44	23.5
>8	89	47.6
Previous history of snake bite		
Yes	35	18.7
No	152	81.3
Site of snakebite		
Leg	113	60.4
Hand	70	37.4
Others	4	2.2
Snake type		
Carpet Viper	164	87.7
Puff adder	5	2.7
Cobra	4	2.1
Not seen	14	7.5
Where was patient when bitten by snake		
At home	24	12.8
At the farm	128	68.4
At the stream	4	2.1
Bush path	31	16.6
Classification of depression		
Mild	25	13.4
Moderate	14	7.5
Severe	8	4.3
No depression	140	74.9
Treatment cost		
>14,400	46	24.6
<14,400	141	75.4
Any snakebite complication		
Yes	25	13.4
No	162	86.6

A total of 47 (25%) had a diagnosis of depression with none recognized by attending clinicians. The pattern of the depression showed that 25 (13.4%) had mild depression, 14 (7.5%) had moderate depression and eight (4.3%) had severe depression (Table 1).

Patients with snakebite associated complications (odd ratio [OR] 3.1, 95% CI 1.1–8.5), and patients with past history of snakebites (different from the index presentation) (OR 2.7, 95% CI 1.1–6.1) were more likely to have mild depression while being a herdsman was protective (OR 0.1, 95% CI 0.01–0.8) (Table 2).

**Table 2:** Factors associated with depression among patients in snakebite wards, General Hospital Kaltungo, 2016

Characteristics^a^	Mild depression		OR	95% CI		P value
	Yes	No				
Past household history of snakebite						
Yes	11 (22.4)	38 (77.6)	2.7	1.1	6.11	0.05
No	14 (10.1)	124 (89.9)				
Worried about time loss						
Yes	3 (5.2)	55 (94.8)	0.3	0.1	0.93	0.05
No	22 (17.1)	107 (82.9)				
Herdsmen						
Yes	1 (2.0)	48 (98.0)	0.1	0.01	0.75	0.01
No	24 (17.4)	114 (82.6)				
Student						
Yes	4 (33.3)	8 (66.7)	3.7	1.02	13.24	0.09
No	21 (12.0)	154 (88.0)				
Had complication following snake bite						
Yes	7 (28.0)	18 (72.0)	3.1	1.14	8.47	0.05
No	18 (11.1)	144 (88.9)				

Moderate depression						
	Yes	No	Mean diff	95% CI		P value
Total treatment cost Mean (Std. Dev)	19,082.9 (10,174.5)	11,332.5 (11,298.9)	7750.3	1681.5	13,819.2	0.02

Severe depression						
	Yes	No	OR or Mean diff	95% CI	P value	
Worried about financial loss						
Yes	7 (10.8)	58 (89.2)	14.6	1.75	121.49	<0.00
No	1 (0.8)	121 (99.2)				
Worried about time loss						
Yes	6 (10.3)	52 (89.7)	7.3	1.43	37.49	0.02
No	2 (1.6)	127 (98.4)				
Worried about family welfare (feeding)						
Yes	5 (35.7)	9 (64.3)	31.5	6.48	152.93	<0.00
No	3 (1.7)	170 (98.3)				
Past history of snakebite						
Yes	5 (14.3)	30 (85.7)	8.3	1.88	36.51	<0.00
No	3 (2.0)	149 (98.0)				
Mean household monthly income (Std. dev)	11,272 (10,992)	36,341 (41,613)	−25,069	−35,509	−14,630	<0.00

^a^ Only statistically significant characteristics shown

Snakebite patients who were moderately depressed spent on the average 7750 Naira (25 USD) more, on total treatment cost and this difference was statistically significant (Mean difference 7750 [25 USD], 95% CI 1681.5 [5.6 USD]–13,819.2 [46.0]) (Table 2).

The following characteristics were significantly associated with severe depression: worrying about family welfare (OR 31.5, 95% CI 6.5–152.9), past history of snakebites (OR 8.3, 95% CI 1.9–36.5), worrying about financial loss (OR 14.6, 95% CI 1.8–121.5) and worrying about time loss (OR 14.6, 95% CI 1.8–121.5). The mean monthly income of snakebite patients with severe depression was significantly lower than for those without severe depression (Mean difference −25,069[84 USD], 95% CI 35,509 [118 USD]–14,630 [49 USD]) (Table 2).

Independent predictors of mild depression were having a snakebite complication (Adjusted Odds Ratio [AOR] 2.9, 95% CI 1.0–8.0) and a past history of snakebite (AOR 2.4, 95% CI 1.0–5.8). Independent predictors for severe depression were worrying about family welfare (AOR 23.8, 95% CI 4.6–124.4) and a past history of snakebite (AOR 5.6, 95% CI 1.1–29.2) (Table 3)

**Table 3:** Predictors of depression among patients in snakebite wards, General Hospital Kaltungo, 2016

Multivariate regression		95% C.I.		P value
Term	AOR	Lower	Upper	
Mild depression				
Past history of snakebite (household)	2.4	1.0	5.8	0.05
Snake bite complication	2.9	1.0	8.0	0.04
Severe depression				
Worried about family welfare (feeding)	23.8	4.6	124.4	<0.01
Past history of snakebite (patient)	5.6	1.1	29.2	0.04

## Discussion

Our study demonstrated the presence of comorbid depression among patients receiving treatment for snakebite envenomation. We found the prevalence of depression to be 25% among snakebite patients with none of the patients recognized as having depression by the attending clinicians.

We found very few peer reviewed studies on psychological disorders following snakebite envenomation worldwide and none at all in Nigeria.

The prevalence of depression (25%) in this study is lower than 50% reported by a similar study of psychological morbidity associated with snakebite envenoming [4]. The difference may be explained by their emphasis on delayed psychological morbidity. The prevalence of depression among the patients is higher than 1.1% prevalence of depression in a community survey in Nigeria [12]. The prevalence of 4.3% for severe depression in this study is higher than 2.5% reported among patients attending a general hospital in a similar cultural setting [13]. The attending physicians were unable to recognize depression in any of the patients despite about 4.3 and 7.5% having severe and moderate depression respectively. This challenge of non-recognition of psychiatric disorders by clinicians practicing in general hospital setting in Nigeria has been previously documented [14, 15]. The lack of integration of psychiatric practice into the lower levels of health care delivery in Nigeria may be responsible. This results in psychiatric services only been available in big tertiary hospitals located in state capitals [16]. Non recognition of psychiatric disorders like depression in general hospitals have been shown to lead to failure of patients to receive appropriate treatment [17, 18], increased cost of health care, length of hospital stay, readmissions and mortality and decreased quality of life [19]. Moreover non-recognition will lead to poor referral of severely ill patients to specialist psychiatric hospitals, the only source of adequate mental health care in most developing countries.

Most of the snakebites patients in this study were farmers/cattle herders (90%) and about 50% were very poor (earn <40 dollars per month) or poor (earn <70 dollars per month). Snakebites has been known to be a problem of the socioeconomically disadvantaged section of our community such as subsistence farmers and herders living in poor rural areas of Nigeria [20].

What is not yet clearly documented is whether these socioeconomic factors will also play a role in the pattern of psychological disorders among this population. In this study, snakebite patients who spent 7750 Naira (26 USD) more, on total treatment cost were more likely to have moderate depression and patients worrying about family welfare, financial and time loss to treatment were more likely to have severe depression. Moreover, the mean monthly income of snakebite patients with severe depression was significantly lower than others. All of the above could indicated that socio-economic factors play a role in the development of depression among the patients. Psychological disorders have been known to be precipitated by adverse life events [21] and it has been suggested that snakebite could be the event that tip the balance leading to development of psychological morbidities [4]. However it is also possible that the presence of the depressive disorders may be responsible for the patient spending more on treatment while on the ward. It has been shown that patients with undetected comorbid psychiatric disorders in general medical settings do spend more on health care cost [19].

Patients who developed snakebite complications were more likely to develop mild depression. Depression is known to be more prevalent among patients with medical conditions [15, 22]. The snakebite complications such as anaemia, wound infections and even limbs amputation could add to the already stressful situation of receiving treatment for snakebite envenomation.

Past history of snakebite (different from the index presentation) was a predictor of both mild and severe depression. This may again be explained by an adverse event (this time happening more than once to the same person) tipping the balance towards the development of psychological disorder. It may also be due to negative connotation given to being bitten by a snake more than once by the community. Other predictors included having a snakebite complication (mild depression) and worrying about relations (severe depression). All of this factors should raise the index of suspicion for depression among attending physicians and should ideally require additional efforts to screening for depression.

This study is a hospital based study and therefore limits the generalization of findings to the community but we are confident that the findings may apply to other snakebites victims with similar socio-cultural factors with patients from the Benue river valley such as Kaltungo.

## Conclusion

We concluded that a quarter of patients admitted for snakebite envenomation in Kaltungo General Hospital had comorbid depression that was unrecognized by the attending clinician. Socioeconomic factors played a role in the development of the depression. Independent predictors of depression such as past history of snakebite, worrying about relations and having snakebite complications could help the attending clinicians anticipate depression among the patients. We recommend training of the clinicians in the hospital on recognition of common psychological disorders like depression; this may include the use of simple depression screening instruments for the detection of comorbid depression.
